# Bact-to-Batch: A Microbiota-Based Tool to Determine Optimal Animal Allocation in Experimental Designs

**DOI:** 10.3390/ijms24097912

**Published:** 2023-04-26

**Authors:** Gaël Even, Anthony Mouray, Nicolas Vandenabeele, Sophie Martel, Sophie Merlin, Ségolène Lebrun-Ruer, Magali Chabé, Christophe Audebert

**Affiliations:** 1 GD Biotech-Gènes Diffusion, F-59000 Lille, France; g.even@genesdiffusion.com (G.E.); s.martel@genesdiffusion.com (S.M.); s.merlin@genesdiffusion.com (S.M.); s.lebrun@genesdiffusion.com (S.L.-R.); 2 PEGASE-Biosciences, Institut Pasteur de Lille, F-59019 Lille, France; 3 Plateforme d’Expérimentations et de Hautes Technologies Animales, Institut Pasteur de Lille, F-59019 Lille, France; anthony.mouray@pasteur-lille.fr (A.M.); nicolas.vandenabeele@pasteur-lille.fr (N.V.); 4 Institut Pasteur de Lille, US 41-UAR 2014-PLBS, Université Lille, CNRS, Inserm, CHU Lille, F-59000 Lille, France; 5 CNRS, Inserm, CHU Lille, Institut Pasteur de Lille, U1019-UMR 9017-CIIL-Centre d’Infection et d’Immunité de Lille, Université de Lille, F-59000 Lille, France

**Keywords:** animal batches, experimental design, microbiota, 16S metagenomics, algorithm

## Abstract

The basis of any animal experimentation begins with the housing of animals that should take into account the need for splitting animals into similar groups. Even if it is generally recommended to use the minimum number of animals necessary to obtain reliable and statistically significant results (3Rs rule), the allocation of animals is currently mostly based on randomness. Since variability in gut microbiota is an important confounding factor in animal experiments, the main objective of this study was to develop a new approach based on 16S rRNA gene sequencing analysis of the gut microbiota of animals participating in an experiment, in order to correctly assign the animals across batches. For this purpose, a pilot study was performed on 20 mouse faecal samples with the aim of establishing two groups of 10 mice as similar as possible in terms of their faecal microbiota fingerprinting assuming that this approach limits future analytical bias and ensures reproducibility. The suggested approach was challenged with previously published data from a third-party study. This new method allows to embrace the unavoidable microbiota variability between animals in order to limit artefacts and to provide an additional assurance for the reproducibility of animal experiments.

## 1. Introduction

The careful design of animal experiments, in particular an optimal allocation method selection, can ensure reliability and reproducibility of experimental studies. Thus, the choice of an appropriate experimental design is of crucial importance, both from an ethical and scientific point of view [1].

Improving the reproducibility of animal experiments is a fundamental condition for complying the guidelines of replacement, reduction, and refinement (3R), in order to reduce the number of animals used and to refine the procedures performed to minimise animal pain and distress [2]. In particular, for reduction, an appropriate design of animal experiments is essential to ensure robust and reproducible results, while maximising the information collected for each animal to avoid the use of additional ones [3].

There are many reasons for poor reproducibility of animal experiments, such as fallacies in experimental design or statistical analyses. Promising specific guidelines and tools, such as the TOP (Transparency and Openness Promotion), the EDA from NC3Rs, ARRIVE or PREPARE guidelines, have thus been developed [4,5,6,7,8]. However, the use of carefully planned and well reported protocols does not automatically guarantee reproducibility. Indeed, observations obtained following the implementation of an experimental design can be submitted to the influence of many uncontrollable environmental background factors that can affect the outcome of the experiment [9].

Variation in the gut microbiome, both within and between experimental groups, is a long-ignored factor that contributes to this irreproducibility [10,11]. Indeed, it is well known that the gut microbiota is an important factor broadly influencing physiology and playing a significant role in human and animal health [12]. For instance, the intestinal microbiota is a key player in the development of the immune system and mucosa [13]. Moreover, the composition and metabolism of the intestinal microbiota can affect other fundamental host processes including drug metabolism and the nutritional status of the host [14]. Moreover, the use of animal models, such as classic rodent models such as mice (*Mus musculus*), plays an important role in the challenge to move from correlation to causal links in the host–microbiome field [15]. Taking all this into account, the significance of the intestinal microbiota in normal physiological functions needs to be considered for the design and interpretation of experiments using animal models, even in disease models where the microbiome is not under investigation.

Although they provide a controlled setting to study the association between host genetics, environmental factors and microbiota composition, mouse models are not exempt from this microbiota variability [10,16]. Even when using defined genetic backgrounds in laboratory mice, the variability in the composition of their gut microbiota can lead to marked phenotypic variations among experimental animals and animal facilities, which is an important confounding factor [10]. In fact, the indeterminate composition and inter-individual diversity of laboratory mice microbiota remain a limitation despite the efforts to standardise breeding and experimental procedures [15,16].

Some authors have proposed solutions to minimise variations in gut microbiota in animals that will be used in an experimental study [17]. In this regard, the choice of assigning animals to the groups to be compared is particularly of rising concern [1]. Some of the few scientific solutions currently available for allocating animals to their experimental housing are the following: (1) organisation by Student’s test; (2) separation and organisation according to the batches constituted by the animal suppliers from the outset; (3) or application of a random distribution [17]. In order to standardise preclinical studies in the host–microbiome field, a simplified mouse microbiota representative of a Specific and Opportunistic pathogen-free (SOPF) microbiota at the functional level, and a standardised gnotobiotic mouse model (GM15), which phenotypically mimics SOPF mice under standard dietary conditions, have also been developed [18]. Furthermore, Robertson et al. demonstrated that the use of F2 generation littermates is a better method than co-housing for standardising gut microbiota between experimental groups [19].

However, a better solution does not mean the best solution. Thus, the issue is not so much to minimise variations in gut microbiota between animals in different test batches, but rather to find the optimal solution to form these groups as homogeneously as possible in terms of microbiota.

Here, we aimed to propose a new experimental strategy to embrace this unavoidable microbiota variability within a single study and thereby to increase reproducibility. This approach, using 16S metagenomic data to guide the assignment of animals, allows to constitute similar groups based on their faecal microbiota patterns.

A pilot study was performed on 20 mouse faecal samples with the objective of establishing two groups (other variables being otherwise identical) of 10 mice that were as similar as possible in terms of their faecal microbiota fingerprinting. Subsequently, this new approach was challenged with previously published data from a third-party study [20].

## 2. Results and Discussion

In the current study, we developed an algorithm to establish before starting the animal experimentation the distribution of the animals into homogeneous groups in terms of their faecal microbiota.

### 2.1. Pilot Study

The pilot study first consisted in a traditional analysis of the gut bacterial microbiota of twenty 6-week-old male C57BL/6J Specific-pathogen-free (SPF) mice (Janvier-Labs). For this purpose, a high-throughput sequencing of the 16S rRNA gene was performed on DNA extracted from the mouse faecal samples (Supplementary File S1). The data analysis led to the estimation of α- and β-diversity indices for these 20 mice.

Therefore, an innovative algorithm named **Bact-to-Batch** and based on HillDiv diversity measurements, combination of dissimilarity matrices and anti-clustering techniques has been developed and applied to these 20 mice. The aim was to provide all the potential experimental allocation designs that were evaluated in terms of microbiota similarity via a metric using Hill numbers and combining α- and β-diversity metrics. At the end of the pipeline, in order to compute the difference between a biased allocation design and an optimal one, a ranking score was set combining the scores of two α-diversity (Chao1 and Shannon) and β-diversity (Weighted UniFrac) indices of mouse gut microbiota, allowing to choose a solution belonging to the landscape of the most optimal solutions. We decided to label the first 5 percentiles of the ranking scores as “optimal designs”. At the opposite extreme, rank scores above the 95th percentile were labelled “high-risk designs”. Designs falling between the 5th and 95th percentile were considered as “sub-optimal designs”.

**Figure 1:**
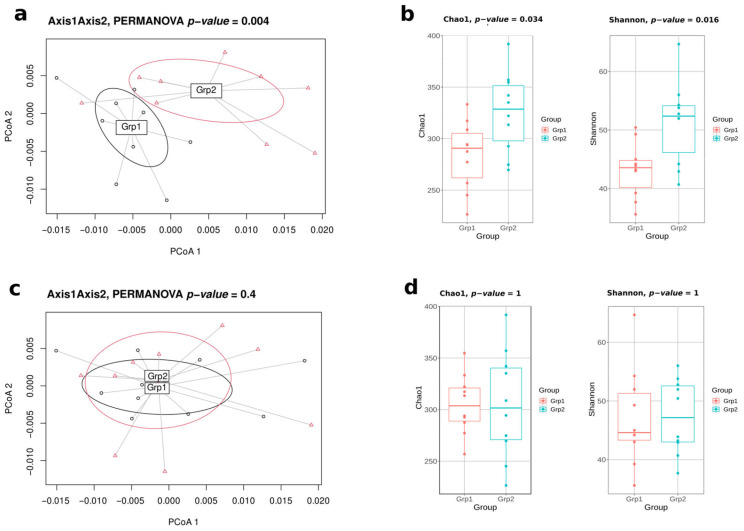
Differences of gut microbiota diversities between the most optimal design obtained by Bact-to-Batch algorithm and the least favourable design of mice allocation in 2 batches of 10. For the least favourable design: (**a**) PCoA displaying two distinct groups in terms of gut microbiota composition using Weighted UniFrac dissimilarities; (**b**) α-diversity boxplots (Chao1 and Shannon indices). For the best favourable design: (**c**) PCoA displaying two non-distinct groups in terms of gut microbiota composition; (**d**) α-diversity boxplots (Chao1 and Shannon indices).

In this pilot study, a naive approach was first tested, consisting of evaluating all the 92,378 possible designs of two batches of 10 mice from the initial 20 ones. For extreme designs (i.e., the optimal design and the least favourable design), it was possible to monitor the biases related to microbiota diversity indices and to highlight the extreme microbiota variability in the least favourable design (Figure 1).

Such an exhaustive approach leading to the calculation of the ranking scores of all the possible designs may be feasible for an allocation of two groups of animals (e.g., a control group vs. a treatment group). For example, in the pilot study, the evaluation of the 92,378 possible designs took 55.4 min in total. However, the number of possible designs and thus the time needed to compute the ranking-score can quickly become a lock (Table 1). For example, the third party study [20] that we used to test our new algorithm, where 28 mice divided into three groups were compared (see below), would have required a computation time of several thousands of years.

Indeed, there is no general formula for partitioning a vector into groups of equal size. The number of possible ways to partition a vector depends on the length of the vector and the size of the groups.

As an illustration, the formula for calculating the number of ways to partition a vector into groups of equal size 2 is given by the following expression: $\frac{n!}{\left(n/k\right)!}$ × *k^(n/k)^* where *n* is the number of elements in the vector, *k* is the size of each group, and ! denotes the factorial function. Another way to approach this issue is to use generating functions, such as the one proposed by the R package RcppAlgos [21].

**Figure 2:**
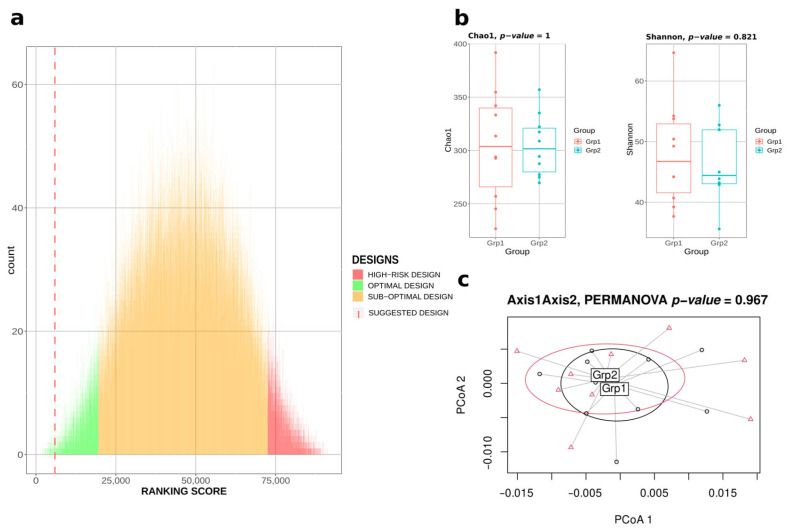
**Bact-to-Batch heuristic approach.** (**a**) Bar chart displaying in green 5% designs maximising the ranking score metric, in red the 5% designs showing a high risk of significant differences in the ranking score, in orange the sub-optimal designs from the 92,378 exhaustive designs. The dotted line corresponds to the optimal suggested design after only 400 iterations of the algorithm. (**b**) Microbiota α-diversity boxplots (Chao1 and Shannon indices) of the two groups of 10 mice from the best suggested design after only 400 iterations. (**c**) PCoA displaying the microbiota β-diversity of the two groups of mice belonging to the best suggested design after only 400 iterations.

Thus, in this paper, we also propose another approach, heuristic instead of exhaustive, involving the sampling of 90,000 possible designs at most to compute the ranking-score. This operation then takes at most one hour with this configuration. To validate this heuristic approach, we even tested the pipeline with only 400 iterations (Figure 2). Satisfyingly, the ranking-score proposed by the algorithm was the 133rd/92,378 (top 0.14%), thus being among the most optimal designs (i.e., green designs in Figure 2a), and the computation time was 13.4 s, representing 0.007% compared to the computation time of the exhaustive approach. Figure 2b,c highlight the similarity of the two groups of mice designed in terms of α-diversity metrics and composition.

**Figure 3:**
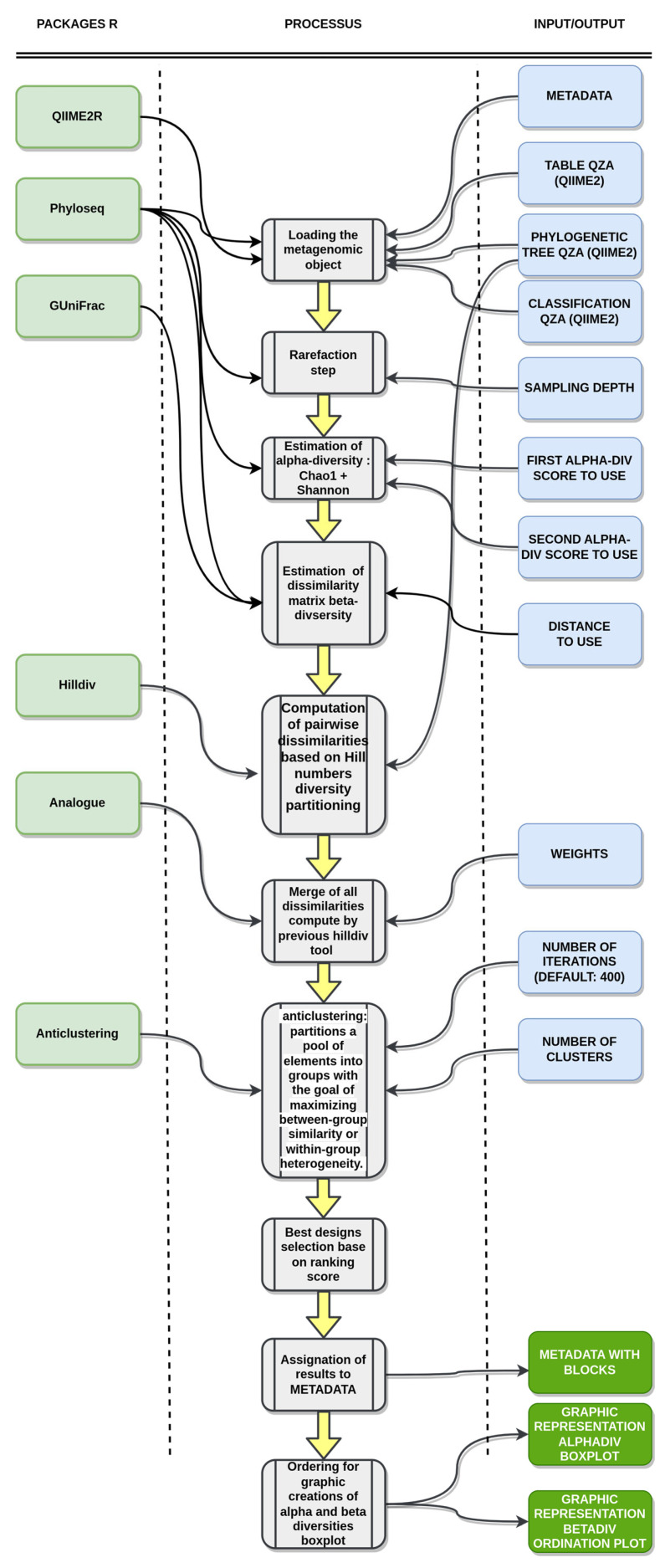
**Bact-to-Batch bioinformatics pipeline**. The pipeline processes mouse gut microbiota profiles as input and provides the constitution of similar animal groups as output, based on gut microbiota fingerprints [22,23,24,25,26,27].

The whole **Bact-to-Batch** bioinformatics pipeline is illustrated in Figure 3.

### 2.2. Validation of the Pipeline with Third Party Data

To validate our heuristic approach, previously available published data were tested with our new algorithm. Only criteria related to sample types (murine faeces), data availability, and metadata completeness were used to determine the study selection [20]. The purpose of the selected study was to examine the tuftsin-phosphorylcholine conjugate (TPC) effect on the gut microbiome in a mouse model of lupus [20]. The authors investigated and compared the gut microbiota of 28 mice divided into 3 groups of 9 to 10 mice (subcutaneously injected with TPC, tuftsin, or phosphate-buffered saline).

**Figure 4:**
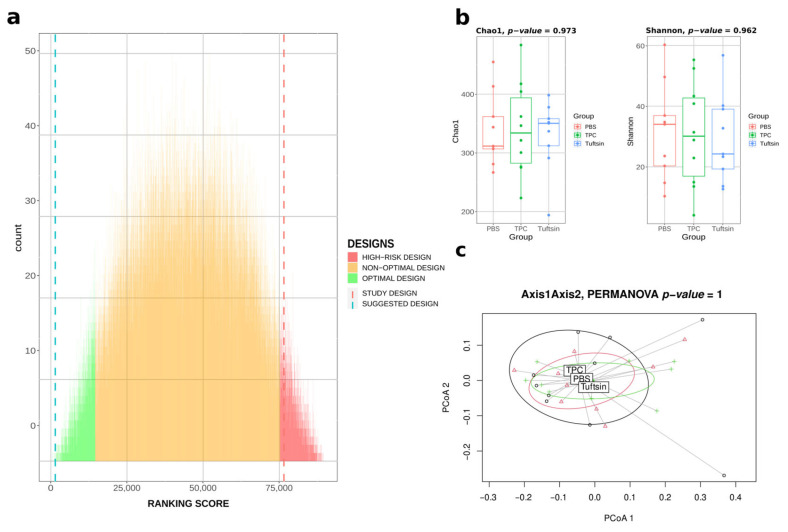
**Bact-to-Batch heuristic approach on third party data.** (**a**) Bar chart displaying the ranking score metrics of 90,000 randomly simulated experimental mice allocation designs (integrating the design chosen during the study of Neuman et al.) obtained by **Bact-to-Batch** algorithm. In green are the 5% optimal designs maximising the ranking score metric, in red are the high-risk designs showing significant differences in the ranking score, in orange the sub-optimal designs. The suggested best design of mice allocation in 3 groups proposed by **Bact-to-Batch** is represented by the blue dotted line and the selected design from the Neuman et al. study by the red dotted line. (**b**) Microbiota α-diversity boxplots (Chao1 and Shannon indices) of the three groups of mice according to our suggested design from **Bact-to-Batch**. (**c**) PCoA displaying the microbiota β-diversity of the three groups of mice belonging to our suggested design from **Bact-to-Batch**.

We used the available metagenomic data of the 28 mice at t = 0 (before any intervention on mice) from the Neuman et al. study to test our **Bact-to-Batch** algorithm using the heuristic approach with 90,000 designs. The mice allocation ranking score in 3 homogeneous groups proposed by our algorithm (Figure 4) is the 6th/90,000 combinations (top 0.007%), while the computation time represents 0.000001% compared to the computation time of the exhaustive analysis. According to our algorithm, the mice allocation that was selected by the authors in their study, most certainly based on randomization, obtained the ranking score of 86,350th/90,000 combinations, i.e., above the 95th percentile, considered in our study as the limit of the high-risk design category (see Supplementary Figure S3 in Supplementary File S1 for α- and β-diversity indices).

Moreover, a differential abundance analysis test with ANCOM-BC global test was performed to check for the presence of significant differentially abundant taxa between the gut microbiota from three groups of mice selected from the Neuman et al. study and the gut microbiota from the three groups of animals determined by **Bact-to-Batch**, assuming that there should be no difference in relative abundances between taxa at t = 0. We found that the **Bact-to-Batch** optimal design resulted in no differentially abundant taxa (0/195, 0%) while the study design by Neuman et al. resulted in 18/195 (9%) significant differentially abundant taxa.

Thus, while not questioning at all the results of these authors’ study, we highlighted here that according to our algorithm, the mice allocation, which was further tested, was not optimal in terms of the gut microbiota homogeneity.

## 3. Materials and Methods

**Faecal sample collection.** Faecal samples were collected from twenty 6-week-old male C57BL/6J Rj Specific-Pathogen-Free (SPF) mice (Janvier-Labs, Le Genest-Saint-Isle, France) that were fed and maintained at the SPF facility of the Institut Pasteur de Lille (research accreditation number: E 59 350 009). On their arrival day at the animal facility, fresh faecal pellets of the 20 mice (two pellets approximately equivalent to 45 mg for each mouse) were collected into sterile tubes under a Biosafety cabinet and then immediately stored at −20 °C until processing. Mice were distributed by the supplier in 7 cages at their arrival. Thus, 3 mice were collected from 6 cages and 2 mice were selected from the 7th cage. The experiment was conducted in accordance with EU Directive 2010/63/10 of 22 September 2010.

**DNA extraction, library preparation and sequencing.** Each sample stored at −20 °C was placed 5 min at room temperature. The extraction was performed by the Nucleospin^®^ 96 Soil kit (Macherey Nagel, Hœrdt, France) under aseptic conditions at room temperature. First, 700 µL of lysis buffer (suitable for samples with organic carbon) was added to rehydrate the sample for 5 min. Then, samples containing the lysis buffer were transferred in tubes containing the supplied beads. DNA extraction was performed by adding Enhancer buffer (allowing for a better yield) in accordance with the supplier’s instructions. The tubes were then subjected to intense 30 Hz agitation for 2 min with bead mills (Retsch, Haan, Germany) to mechanically lyse the samples. The protocol followed the supplier’s recommendations until the elution phase that occurred after incubation of the plates with 50 µL of TE 1X preheated at 70 °C followed by a 1-h incubation at room temperature and finally an elution phase after centrifugation at 6000× *g* for 2 min.

To achieve the sequencing library, which is based on the principle of dual-indexing thanks to paired-end sequencing, two PCRs were successively applied: from 2 µL of the extracted DNA diluted to 1/200, a first PCR in final volume of 50 µL, 1 U of Phanta Max Super-Fidelity DNA Polymerase (Vazyme, CliniSciences, Nanterre, France), each primer has a final concentration of 500 nM. For this first PCR, forward and reverse primers have been designed with a 5′-Tag sequence, respectively, 5′-TCGTCGGCAGCGTCAGATGTGTATAAGAGACAG-3′ for forward primer and 5′-GTCTCGTGGGCTCGGAGATGTGTATAAGAGACAG-3′ for reverse primer and a 16S rRNA gene specific sequence, respectively, 5′-CCTACGGGNGGCWGCAG-3′ for forward primer and 5′-GACTACHVGGGTATCTAATCC-3′ for reverse. According to *E. coli* 16S rRNA sequence gene, specific primers target a locus between position 341 and 785, resulting in the amplification of a locus of 445 bp. The amplification conditions were 3 min at 95 °C, 25 cycles of 15 s at 94 °C for denaturation, 15 s at 51 °C for primers annealing and 45 s at 72 °C for extension, followed by an incubation at 72 °C for 1 min. At the end of this first PCR, amplification products were purified with NucleoFast^®^ 96 PCR (Macherey Nagel) according to the supplier’s recommendations except for the last step for which 30 µL of TE 1X preheated at 70 °C have been used for elution. From 5 µL of the previously purified DNA, a second PCR was performed in a final volume of 50 µL, 1 U of Phanta Max Super-Fidelity DNA Polymerase (Vazyme, CliniSciences). A final concentration of both primers at 500 nM was used. The amplification conditions were the same as those of the previous one except the number of cycles reduced to 8. In addition to the Tag sequences, these PCR2 primers contained a locus to index the samples (barcode sequence) and a locus sequence adapter suitable for the Illumina sequencing technology. A NucleoFast^®^ (Macherey Nagel) purification step identical to the one presented above was performed followed by a Quant-iT PicoGreen ds DNA quantification (Life Technologies, Carlsbad, CA, USA). An equimolar pool of libraries was produced and 200 µL of this mixture was purified by NucleomagNGS^®^ (Macherey Nagel). The purification was performed twice with 1.2 X of beads suspension according to the supplier’s instructions to conclude with a final elution in 50 µL of TE 1X pH 8.0. This purified mixture was diluted by 10 and by 20 and then monitored by Bioanalyzer high sensitivity assay (Agilent Technologies, Santa Clara, CA, USA) as a quality control to validate the profile and obtain the average size. Then, a quantification of DNA was realised by Qubit High Sensitivity assay (Invitrogen, Carlsbad, CA, USA). The concentration of DNA and the average size were used to assess molarity of the purified mixture.

Sequencing library has been paired-end sequenced on MiSeq platform (Illumina, Évry-Courcouronnes, France) with MiSeq Reagent Kit v3 allowing 600 sequencing cycles to be performed. At the end of the sequencing a quality control by FastQC was carried out.

**Data processing and statistical analysis.** After raw amplicon reads generation, QIIME2 [22] microbiome analysis package was used to process the samples. Briefly, raw fastq files were first converted into QIIME2 compatible files. After a quality check, DADA2 [28] was used to generate an Amplicon Sequence Variant (ASV) table and their respective representative sequences. De novo phylogenetic trees used to calculate phylogenetic distance and diversity were also built using default QIIME2 provided methods, i.e., align-to-tree-mafft-fastree (MAFFTm multiple sequence alignment program) [29,30].

SILVA database v138 [31] was used as the reference database in combination with QIIME2′s pre-fitted sklearn-based taxonomy classifier tool for ASV taxonomic classification.

For computation of pairwise dissimilarities among samples, the function *pair_dis* from hilldiv package [25] was used for generation of pairwise dissimilarity matrices from count tables using Hilldiv diversity measure. Hilldiv diversity is based on the Hill numbers, which are a family of diversity indices that were first proposed by the ecologist Robert Hill in 1973. Hill numbers provide a measure of the diversity of a community based on the number and relative abundance of its species or other units of diversity. Hilldiv diversity is calculated using the formula:

Hilldiv=1∑pi^q

where *pi* is the relative abundance of each species or unit of diversity, and *q* is a parameter that determines the sensitivity of the diversity measure to changes in the relative abundance of the species. *Hilldiv* diversity is often used in metagenomic studies to compare the diversity of different microbial communities and to assess the impact of different factors on microbial diversity. A-diversity is obtained by computing the Hill numbers from the averaged basic sums of the samples, while γ-diversity is obtained by taking the average of OTU relative abundances across samples, and then computing the Hill numbers of the pooled system. The division of γ-diversity by α-diversity yields the β-diversity, which quantifies how many times richer an entire system is in effective features (γ-diversity) than its constituent samples are on average (α-diversity). Four types of dissimilarity matrices were computed using different measures of similarity: Sørensen-type overlap, Jaccard-type overlap, Sørensen-type turnover, and Jaccard-type turnover. A combined dissimilarity matrix was then computed using the fuse function from analogue R package [26]. Fusing dissimilarity matrices is a technique used in data analysis to combine multiple dissimilarity matrices into a single matrix. To “fuse” dissimilarity matrices, the individual matrices are first standardised to ensure that they are on the same scale, and then they are combined using an even weighted average.

The resulting fused matrix was then used for selecting homogeneous groups using anti-clustering function from anticlust R package [27]. Anti-clustering is a term used in data mining to refer to the opposite of clustering. Anti-clustering partitions a pool of elements into groups with the goal of maximising between-group similarity or within-group heterogeneity. The anti-clustering approach thereby reverses the logic of cluster analysis that strives for high within-group homogeneity and low similarity of the different groups.

In order to select the best design, a ranking score was set combining two α-diversity scores (Chao1 and Shannon, considered respectively as Richness and Evenness measures) and β-diversity (Weighted unifrac) with 50% weight on α-diversity and 50% weight on β-diversity. All graphical outputs were generated using the ggplot2 R package [32]. A-diversity was computed using the phyloseq R package [23] and β-diversity dissimilarities matrix were computed using the GUniFrac R package [24]. Multivariate dispersions for Principal Coordinate Analysis (PCoA) axes generation were computed using *betadisper* function from vegan package [33].

ANCOM-BC v1.6.1 [34] (Analysis of Composition with Benjamini-Hochberg Correction) statistical test was used to identify differentially abundant species in microbial communities.

All graphical outputs were generated using the ggplot2 R package. The experiments were conducted on a DELL PowerEdge R640 Server equipped with a 3.6 GHz Intel(R) Xeon(R) Gold 6244 processor, 1024 GB of DDR4 RAM, and a 14 TB solid state drive. The workstation ran the Ubuntu 20.04.4 LTS operating system, and the experiments were implemented in R language.

## 4. Conclusions

Since the gut microbiota is recognized to have a comprehensive influence on animal experiments results (whether or not these experiments are based on a metagenomic study), we proposed a new approach named **Bact-to-Batch** that will allow to constitute homogeneous groups of animals to be compared, in terms of their microbiota fingerprinting, prior to the experiment. This approach emphasises the value of relying on biological data collected non-invasively (such as the faecal microbiota) to supervise the achievement of animal batches. This new method will limit artefacts and provide an additional guarantee for reproducibility and greater robustness in the analysis of animal test results. The implementation of this new approach may present some drawbacks, such as the availability and time to perform high throughput sequencing if it is obtained by a second-generation instrument, but we are convinced that these inconveniences can be rapidly overcome by the use of third generation real-time sequencers. Thus, as soon as they arrive in the laboratory, it seems quite feasible to test the animals and run the **Bact-to-Batch** algorithm to obtain the best allocation solution in a very short time.

## Figures and Tables

**Table 1:** Evolution of the computation time of our algorithm according to the number of possible designs related to the number of animals and the number of batches to constitute (3.6 GHz Intel(R) Xeon(R) Gold 6244 processor, 1024 GB of DDR4 RAM, and a 14 TB solid state drive).

*n* Animals	*n* Groups	*n* Possible Designs	CPU Time (s)	CPU Time (Year)
10	2	1.26 × 10^2^	4.50	1.43 × 10^−7^
12	2	4.62 × 10^2^	1.66 × 10^1^	5.26 × 10^−7^
12	3	5.78 × 10^3^	2.08 × 10^2^	6.58 × 10^−6^
20	2	9.24 × 10^4^	3.32 × 10^3^	1.05 × 10^−4^
20	4	4.89 × 10^8^	1.06 × 10^9^	3.35 × 10^1^
24	2	1.35 × 10^6^	2.92 × 10^6^	9.25 × 10^−2^
24	3	1.58 × 10^9^	3.41 × 10^9^	1.08 × 10^2^
27	3	3.80 × 10^10^	8.20 × 10^10^	2.60 × 10^3^
28	4	1.97 × 10^13^	4.25 × 10^13^	1.35 × 10^6^
30	2	7.76 × 10^7^	1.67 × 10^8^	5.30
30	3	9.25 × 10^11^	2.00 × 10^12^	6.33 × 10^4^

## Data Availability

A code repository has been created for the bioinformatics analysis and is available at https://github.com/GEbiotech/Bact-to-Batch (private access at this step). All meta-analysis and sequencing data used for the third party experiment [20] were obtained from publicly accessible databases (https://www.ebi.ac.uk/ena/browser/view/PRJEB24885 (accessed on 1 February 2023)).

## Supplementary Materials

The following supporting information can be downloaded at: https://www.mdpi.com/article/10.3390/ijms24097912/s1.
