# Supplementary material for: Bact-to-Batch: A Microbiota-Based Tool to Determine Optimal Animal Allocation in Experimental Designs
Source: Int J Mol Sci. 2023 Apr 26;24(9):7912. doi: 10.3390/ijms24097912 (PMC10178137; doi:10.3390/ijms24097912)
Supplement: Supplementary file 1 [file ijms-24-07912-s001.zip › ijms-2345423-supplementary.pdf]

sequence counts summary

Minimum:	48639
Median:	98827.5
Mean:	98799.5
Maximum:	215012
Total:	1975987

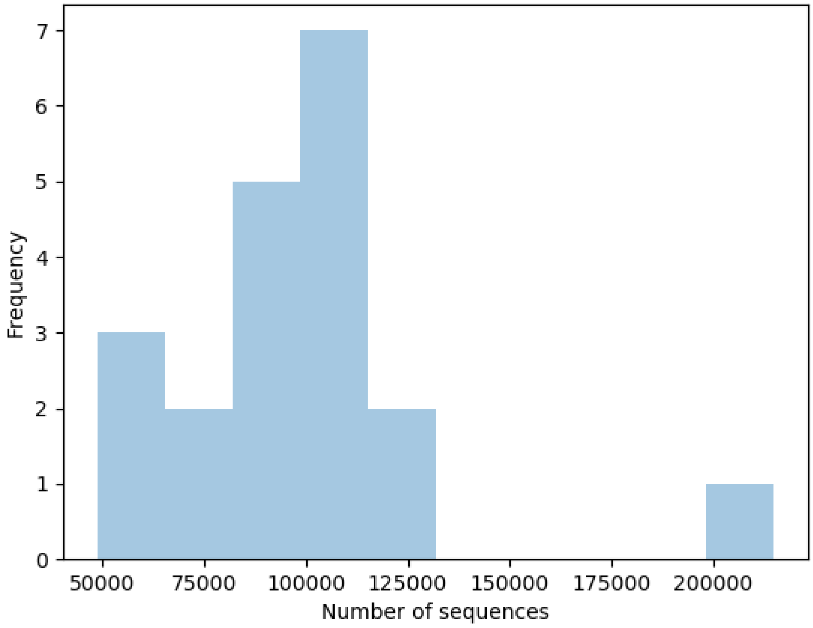

Supplementary Figure S1 | Sequencing data sets generated in this study.

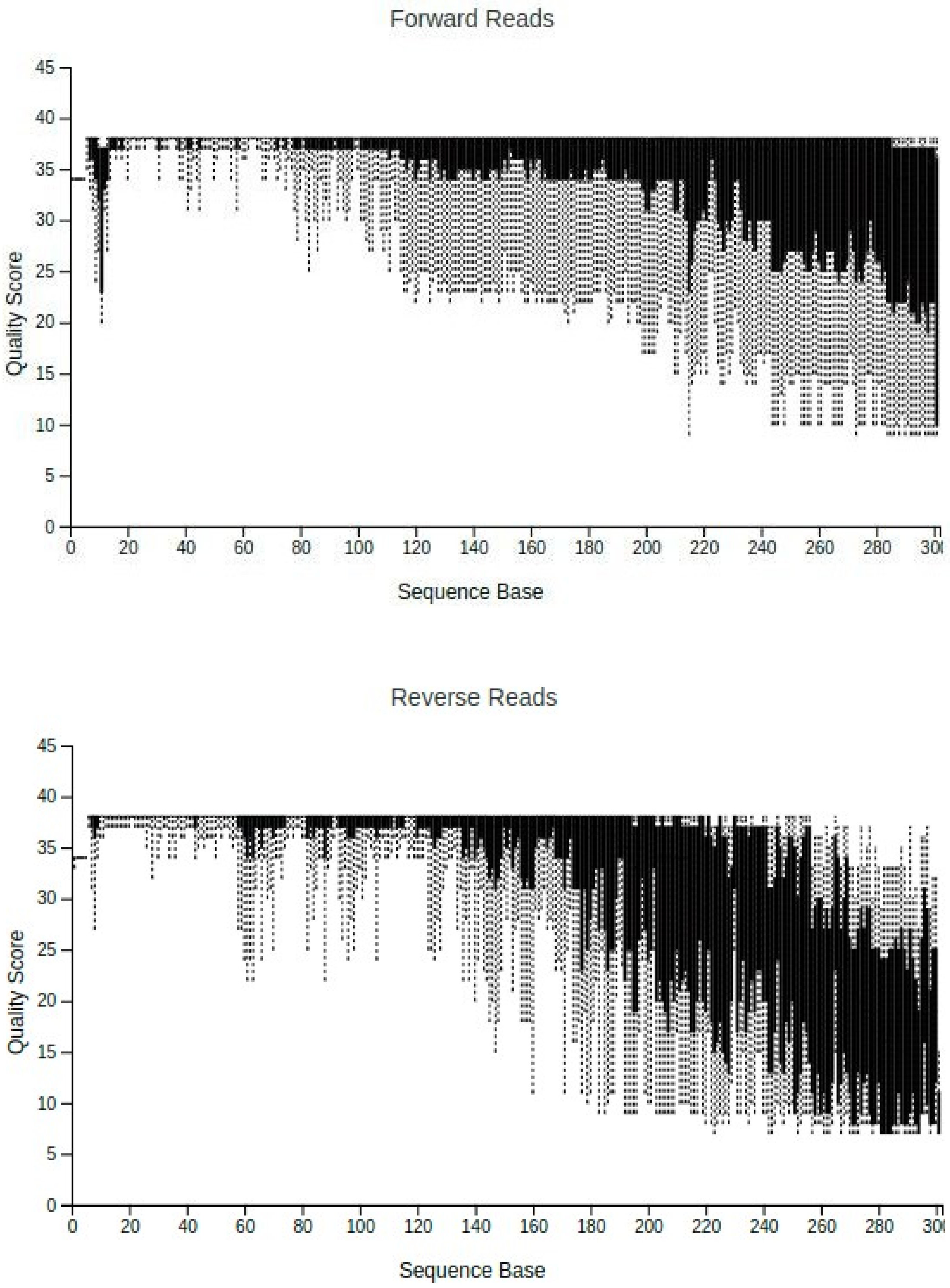

Supplementary Figure S2 | Quality plots for forward and reverse reads.

SAMPLES NUMBER	NUMBER OF ASVs	MEAN READS BEFORE DADA2	MEAN READS AFTER DADA2	FINAL PERCENTAGE OF RETAINED READS
20	947	94103.7	44753.7	47.1

Supplementary TableS1 | Summary metrics after DADA2 Denoise and dereplicate paired-end sequences.

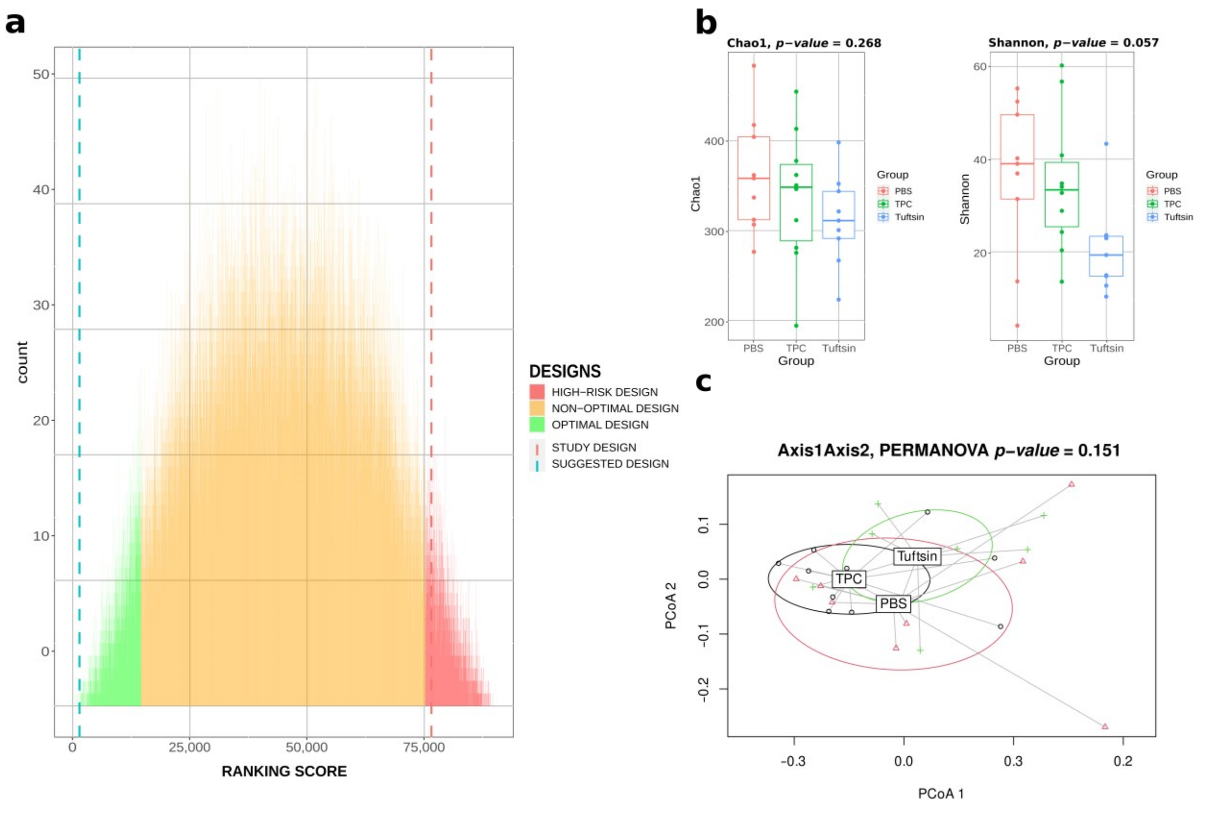

Supplementary Figure S3 | Analysis of the mice allocation design selected by the third party study. a, Bar chart displaying the ranking score metrics of 90,000 randomly simulated experimental mice allocation designs (integrating the design chosen during the study of Neuman *et al.*) obtained by **Bact-to-Batch** algorithm. The suggested best design of mice allocation in 3 groups proposed by **Bact-to-Batch** is represented by the blue dotted line and the selected design from the Neuman *et al.* study by the red dotted line. **b**, Microbiota α-diversity boxplots (Chao1 and Shannon indices) of the three groups of mice according to the Neuman *et al.* study. **c**, PCoA displaying the microbiota β-diversity of the three groups of mice belonging to the Neuman *et al.* study design.
